# Decreased Expression of SATB2: A Novel Independent Prognostic Marker of Worse Outcome in Laryngeal Carcinoma Patients

**DOI:** 10.1371/journal.pone.0040704

**Published:** 2012-07-16

**Authors:** Tian-Run Liu, Li-Hua Xu, An-Kui Yang, Qian Zhong, Ming Song, Man-Zhi Li, Li-Juan Hu, Fu-Jin Chen, Ze-Dong Hu, Ping Han, Mu-Sheng Zeng

**Affiliations:** 1 State Key Laboratory of Oncology in South China, Guangzhou, Guangdong, People’s Republic of China; 2 Department of Otorinolaryngology Head and Neck Surgery, The Sixth Affiliated Hospital of Sun Yat-sen University, Guangzhou, Guangdong, People’s Republic of China; 3 Department of Experimental Research, Sun Yat-sen University Cancer Center, Guangzhou, Guangdong, People’s Republic of China; 4 Department of Head and Neck Surgery, Sun Yat-sen University Cancer Center, Guangzhou, Guangdong, People’s Republic of China; 5 Department of Head and Neck Surgery, The Third Affiliated Hospital of Kunming Medical University, Kunming, People’s Republic of China; 6 Department of Otorhinolaryngology-Head and Neck Surgery, Sun Yat-sen Memorial Hospital, Guangzhou, Guangdong, People’s Republic of China; The University of Hong Kong, China

## Abstract

**Background:**

To investigate the expression and role of special AT-rich sequence-binding protein-2 (SATB2) in laryngeal squamous cell carcinoma (LSCC) tissue and cell line (HEp2), and to evaluate the clinical and prognostic significance of SATB2 protein in patients with LSCC.

**Methods:**

The expression of SATB2 was examined in LSCC tissue and HEp2 cells by Western-blotting, Real-time PCR and immunohistochemical staining. Cell growth curve assay and colony formation assay were used to verify the effect of SATB2 on the proliferation and tumor progression ability of HEp2 cells. Tumor formation assay in nude mice was used to analyze the effect of SATB2 on the tumorigenicity of HEp2 cells.

**Results:**

The status of SATB2 protein in carcinoma tissues is much lower than that in paracarcinoma tissues. The overall survival of the patients with high SATB2 expression was significantly higher than the low SATB2 expression group. Lower or negative SATB2 expression was significantly correlated with advanced clinical staging, histological grade and tumor recurrence. *In vitro* experiments demonstrated that over-expression of SATB2 in HEp2 cells inhibited cell proliferation and tumor progression ability, and down-regulation of SATB2 showed the opposite effects. Over-expression of SATB2 repressed the tumorigenicity of HEp2 cells by *in vivo* experiments. Moreover, multivariate analysis suggested that SATB2 expression might be an independent prognostic indicator for the survival of LSCC patients after curative surgery.

**Conclusions:**

SATB2 might involve in the development and progression of LSCC as a tumor suppressor, and thereby may be a valuable prognostic marker for LSCC patients.

## Introduction

Laryngeal squamous cell carcinoma (LSCC) is the second most frequent malignancy in head and neck region, accounting for 2% of all human malignancies [1], [2], [3]. Patients with LSCC have a poor survival, which has not been increased over the last 30 years [4]. The molecular mechanisms in the development and progression of LSCC remain poorly understood. Current methods used to predict the outcome of LSCC patients mainly depend on the clinicopathological factors, such as TNM stage, differentiation grade and metastasis [5], [6], [7], [8], [9], [10]. Therefore, other parameters such as molecular markers are needed to more accurately predict the outcome of this disease. It is of great value in further understanding the molecular mechanisms of LSCC and find valuable early molecular diagnostic markers and molecular prognostic factors with high specificity and sensitivity and novel therapeutic strategies.

Human special AT-rich sequence-binding protein-2 (SATB2) is a novel AT-rich DNA binding protein, which is involved in regulating gene expression through altering chromatin structure [11], [12]. SATB2 directly interacts with the activity of transcription factors that regulate craniofacial development and cortical neurons differentiation [13], [14], [15], [16]. SATB2 also regulates skeletal development and osteoblast differentiation, and modulates immunoglobulin gene expression [11], [15]. SATB1, the homologous gene of SATB2, is a nuclear protein with a great role in regulating gene expression during the differentiation and activation of T cells in the immune system [17], [18], [19]. Furthermore, recent study indicated that SATB1 is an essential factor in the aggression of breast cancer, and SATB1 promoted tumor growth and metastasis of breast cancer by regulating the expression of hundreds of genes [20].

Although SATB1 and SATB2 are homologous genes in which their amino acid sequence exhibits 60% homology, and SATB1 is a potential oncogene [20], the role of SATB2 in cancer is still unclear. Until now, limited studies have been done to evaluate the relationship between SATB2 expression and cancer progression, such as colorectal, breast and oral carcinomas [21], [22], [23], [24]. The results of these investigations, however, were highly controversial [21], [22], [23], [24]. In addition, there were no LSCC samples included in the most recent study in head and neck squamous cell carcinomas (HNSCCs) [24]. To explore the exact role of SATB2 in LSCC, we investigated whether the expression of SATB2 protein is different between tumor tissues and normal tissues, whether SATB2 has any role in the development and progression of LSCC, and whether SATB2 is a prognostic factor in LSCC after curative surgical treatment.

## Materials and Methods

### Patients and Specimens

Fresh tumor tissue samples with paired non-cancerous mucosa of 24 LSCC patients were obtained in operation from the Sun Yat-sen University Cancer Center (SYSUCC). A total of 86 paraffin-embedded LSCC samples, which were histologically and clinically diagnosed in patients with radical surgery in SYSUCC, between 1999 and 2002, were also included in this study. Resected specimens, fixed in 10% formalin solution and then embedded in paraffin, were longitudinally sliced into 4-mm-thick sections. Representative sections were prepared and stained with hematoxylin and eosin for histologic examination. Western-blot was used to confirm the specificity of SATB2 staining in fresh LSCC tissues with paired non-cancerous laryngeal mucosa tissues and HEp2 cell line. None of these patients had received radiotherapy or chemotherapy prior to surgical treatment. Clinical and pathological data of the 86 patients with LSCC were collected, such as age, tumor size, stage, differentiation grade, lymph node metastases, treatment and recurrence. The tumor stages were classified according to the 2002 TNM staging system of Union for International Cancer Control (UICC). Clinical follow-up information was obtained by telephone or from the outpatient records.

Written Ethics Approval and Patient Consent from the Sun Yat-sen University Cancer Center Hospital Research Ethics Committee were obtained. Participants were recruited and human experimentation was conducted in Sun Yat-sen University Cancer Center. We have obtained written informed consent from all participants involved in the study.

### Cell Culture

The LSCC cell line HEp2 and human tongue squamous carcinoma cell lines (Tca8113 and TSCCa) were obtained from The Cell Bank of Type Culture Collection of Chinese Academy of Sciences. Cells were maintained in RPMI 1640 medium (Gibco, Invitrogen, Carlsbad, CA) supplemented with 10% fetal bovine serum (FBS; Hyclone, Logan, UT), penicillin (100 units/ml), and streptomycin (100 units/ml) at 37°C in humidified 5% CO_2_ incubator.

### Western Blotting Analysis

Cell and tissue samples were solubilized in SDS lysis buffer, and the protein concentrations were detected by the BCA protein assay kit (PIERCE, Rockford, IL). Equal amounts of protein samples (30 µg/lane) were separated by electrophoresis through 9.0% resolving SDS–polyacrylamide gel, and then transferred to PVDF membranes (Amersham Pharmacia Biotech Inc in Piscataway, NJ). Block the non-specific binding sites by immersing the membrane into 5% non-fat milk in TBS-T solution for 1 hr, and then incubate the membrane with a primary monoclonal antibody to SATB2 (Abcam and ProSCI) for 2 hr at room temperature (RT). After washing 3 times in with TBS-T (TBS+0.5% Tween-20), the membranes were incubated with a secondary antibody (diluted 1∶1000 in TBS-T) for 1 hr at RT. The membranes were washed and proteins were detected by enhanced chemiluminescence system (Amersham Pharmacia Biotech) following manufacturer's instructions. Anti-α-tubulin mouse monoclonal antibody was used to confirm equal loading of lysates (1∶1000; Santa Cruz Biotechnology). Image J software was used to analyze the gray value.

### Real-time RT-PCR Analysis

Total RNA from human tissues and cell lines was extracted using Trizol reagent (Invitrogen) according to the manufacturer’s instructions. cDNA was synthesized from 1 µg of total RNA by use of the SuperScript® III First-Strand Synthesis System (Invitrogen). Real-time PCR was carried out using an CFX96 Real-Time System (BIO-RAD). SYBR green 2X master mixture (Invitrogen) was used in a total volume of 10 μL. The primer sequences were as follows: SATB2 sense 5′- GGAGAACGACAGCGAGGAA-3′, antisense 5′- CCGAT GTATTGCTTTGCCTAGT-3, SATB1 sense 5′-TGCAAAGGTTGCAGCAACCAAAAGC-3′, antisense 5′ -AACATGGATAATGTGGGGCGGCCT-3, GAPDH sense 5′-TGTTGCC ATCAATGACCCCTT-3′, antisense 5′- CTCCACGACGTACTCAGCG-3′, GAPDH was used as an internal control. All reactions were run in triplicate in three independent experiments.

### Immunohistochemical Analysis

Immunohistochemical (IHC) staining was performed using Dako Envision system (Dako, Carpinteria, CA) according to the manufacturer's instructions. The paraffin-embedded specimens were cut into 4 µm sections and baked 1 h at 65°C. All sections were deparaffined with xylenes and rehydrated through graded ethanol series to distilled water. Then, the sections were submerged into EDTA antigenic retrieval buffer (pH 8.0) and microwaved for antigenic retrieval. The sections were treated with 0.3% H_2_O_2_ for 15 min to block the endogenous peroxidase at RT, and then were treated with normal goat serum for 30 min to reduce the nonspecific binding. Consequently, the sections were incubated with rabbit polyclonal anti-SATB2 antibody (1∶200; Bethyl Laboratories) overnight at 4°C. After washing, the sections were incubated with biotinylated anti-rabbit secondary antibody (Zymed) followed by further incubation with streptavidin-horseradish peroxidase (Zymed) at 37°C for 30 min. Diaminobenzidine (DAB) was used for color reaction, and the antibody was replaced by normal goat serum for negative controls.

The immunohistochemically stained tissue sections were scored independently by two pathologists blinded to the clinical parameters, and the final score was the average of the scores by two observers. We used the intensity and extent of the staining to evaluate the expression of SATB2. The staining intensity was scored as 0 (no staining), 1 (weak staining exhibited as light yellow), 2 (moderate staining exhibited as yellow brown), 3 (strong staining exhibited as brown). Extent of staining was scored as 0 (0%), 1 (1 to 25%), 2 (26 to 50%), 3 (51 to 75%), and 4 (76 to 100%), according to the percentages of the positive staining areas relative to the whole carcinoma area or entire section for the normal samples. The sum of intensity and extent score was used as the final staining scores (0 to 7) for SATB2. For the purpose of statistical evaluation, tumors having a final staining score of <3 classified tumors with low SATB2 expression and score ≥3 classified as high SATB2 expression. Based on the previous studies [25], the distribution of SATB2 was scored as follows: negative (<10% of the cells being positive) and positive (more than 10% of the cells were positive).

### Plasmid Construction and Retroviral Infection

The plncx2 vector was used to generate plncx2-SATB2. The pSUPER.retro.puro vector was used to generate pSUPER-SATB2 shRNA. Production of retrovirus was performed according to the instructions, HEp2 cells and Tca8113 cells were subjected with infection of retrovirus expressing SATB2 or plncx2 (empty vector) and HEp2 were also used to knock-down the expression of SATB2. HEp2 cells or Tca8113 cells expressing SATB2 or empty vector were selected for 10 days with G418 after infection. After 10 days selection, the HEp2-SATB2 cells and HEp2-plncx2 cells were verified and cultured in fresh medium. Targeted SATB2 sequence: SATB2-SH1, GGAATAATCAAGCTGGGAA; SATB2-SH2: CCATGGCC CATCTGATAAA; SATB2-SH3: GCGGAGCATGAATCCCAT.

### Colony Formation Assay

For colony formation assay, cells were seeded evenly in 6-well plates (2×10^2^ cells per well) and cultured for 14 days. Then the cells were fixed with methanol for 10 min, stained with 1% crystal violet for 1 min. Each group of cells was performed in triplicate.

### 3-(4,5-dimethylthiazol-2-yl)-2,5-diphenyltetrazolium Bromide Reduction (MTT) Assay

Cells were seeded onto 96-well plates at 2000 cells/well. Each sample had four replicates. The cells were incubated with 0.2% MTT for 4 h at 37°C, 100 ul DMSO/well was added to the culture cells to dissolve the crystals, and cells were counted every day by reading the absorbance at 490 nm.

### Annexin V Flow Cytometry Assay

Apoptosis was measured with the Annexin V-FITC Apoptosis Detection KIT (invitrogen) according to the instructions and analyzed by flow cytometry (Epics Elite, Beckman Coulter, Brea, CA).

### Tumor Formation in an Animal Model

Equivalent amounts of HEp2-plncx2 or HEp2-SATB2 cells (5 × 10^5^ cells) were injected subcutaneously into the right flank of female BALB/c nude mice (Shanghai Slac Laboratory Animal Co. Ltd, Shanghai, China) at 5 weeks of age (16–18.5 g). Tumorigenesis procedure was observed by measuring solid tumors in 2 dimensions with a caliper for 23 days. Animals were sacrificed 23 days after injection. The experiments on mice had been approved by the ethics committee at SYSUCC.

### Statistical Analysis

Statistical analyses were performed using a statistical software package (SPSS13.0, Chicago, IL). The significance of SATB2 or SATB1 mRNA levels was determined by t-test. The chi-square test was used to analyze the relationship between SATB2 expression and clinicopathological characteristics. Survival times were evaluated using the Kaplan and Meier survival curves, and compared by the log-rank test. The significance of various variables for survival was analyzed by multivariate survival analysis using Cox's regression model. *P*-value less than or equal to 5 percent were considered to be statistically significant.

## Results

### The Expression of SATB2 in LSCC Tissues

**Figure 1:**
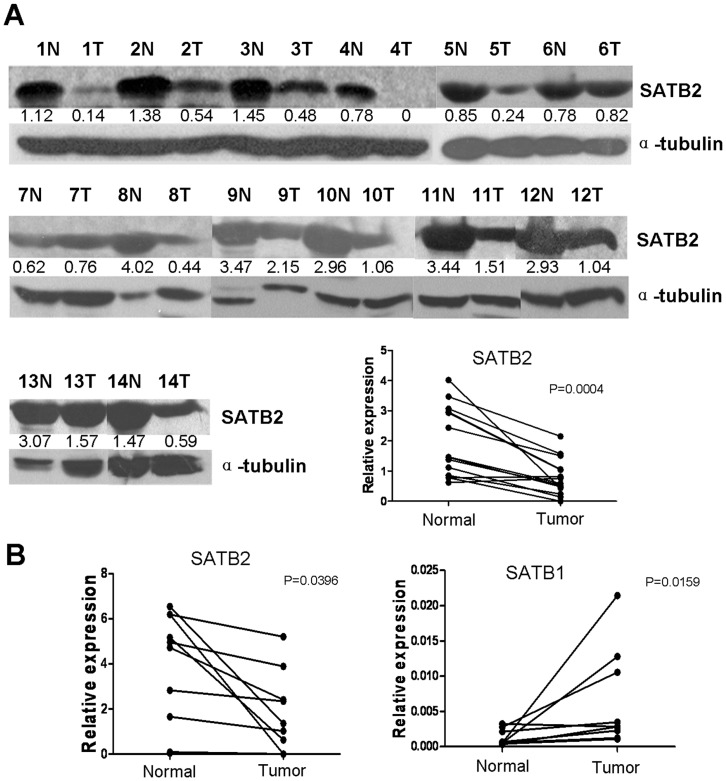
Expression levels of SATB2 and SATB1 in laryngeal squamous cell carcinoma tissues. A. Expression levels and quantitative analysis of SATB2 protein in 14 paired laryngeal squamous cell carcinoma tissues by Western blotting. N, paracarcinoma (normal) laryngeal epithelial tissues. T, laryngeal squamous cell carcinoma tissues. The numbers under each SATB2 lane indicate related expression of each sample by compared the densitometry of the SATB2 and the internal control. B. mRNA levels of SATB2 and SATB1 in 10 paired laryngeal squamous cell carcinoma tissues by real-time PCR.

To determine the expression of SATB2 protein in LSCC tissues, Western blotting was performed in 14 LSCC tissues with paired non-cancerous mucosa. Among 12 of 14 LSCC tissues with paired normal mucosa, clearly decreased levels of SATB2 expression was detected in all the tumors tissues in comparison to the paired non-cancerous mucosal tissues (Figure 1A). However, the levels of SATB2 expression were similar in both tumors tissues and non-cancerous mucosal tissues in the rest 2 paired LSCC tissues (Figure 1A, patient samples No. 6 and No. 7). We then determined whether the reduced expression of SATB2 occurred at mRNA level. We obtained an extra 10 paired LSCC samples for real-time RT-PCR analysis. As shown in Figure 1B, the expression level of SATB2 mRNA is significantly lower in tumor tissues. However, the expression of SATB1, a close homologue of SATB2, is significantly upregulated in most of these tumor samples. These data suggested that unlike the oncogenic role of SATB1 [20], SATB2 might serve as a tumor suppressor gene in LSCC.

**Figure 2:**
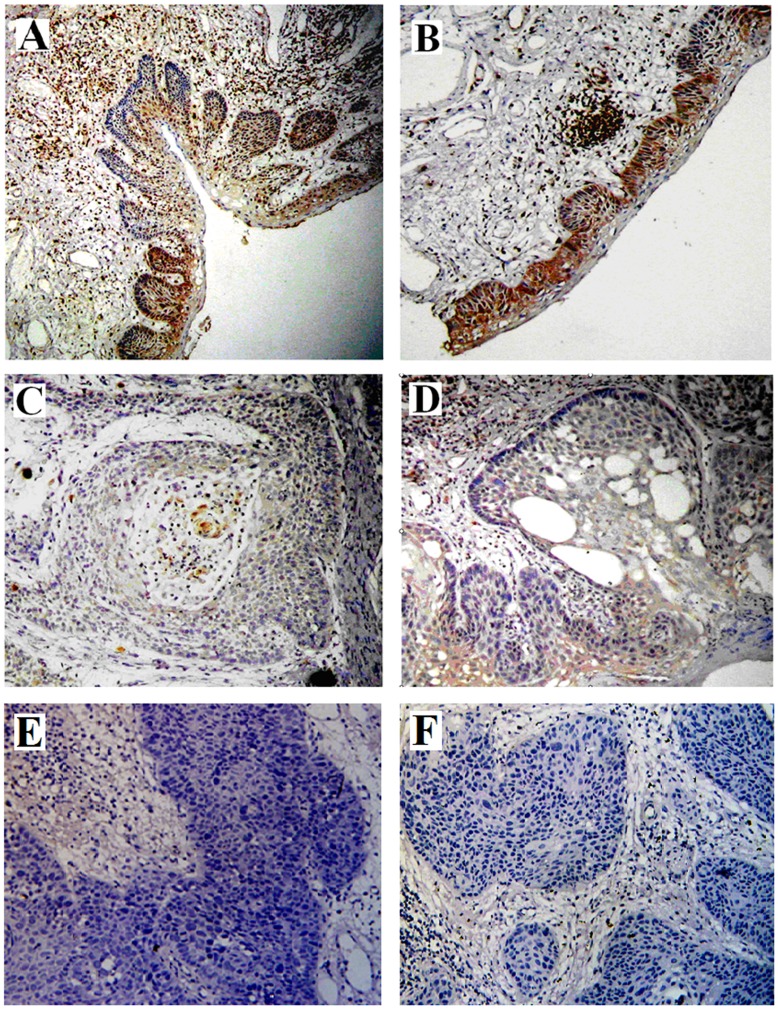
Analysis of SATB2 protein in tissues by immunohistochemistry. A and B, SATB2 expression is strong in normal laryngeal epithelial cells. C and D, SATB2 expression is weak in well-differentiated laryngeal squamous cell carcinoma cells. E and F, SATB2 expression is negative in poor-differentiated laryngeal squamous cell carcinoma cells. (×200).

To verify this observation, we further examined the expression of SATB2 protein in 86 paraffin-embedded LSCC samples and 15 normal laryngeal epithelial (non-cancerous) samples by immunohistochemical analysis (Figure 2). As shown by immunohistochemical analysis, 38 of 86 (44.2%) paraffin-embedded laryngeal cancer tissues showed negative nuclear or cytoplasm staining of SATB2 protein, while 37 of 86 (43.0%) laryngeal cancer tissues showed mainly moderate SATB2 staining (in the nuclear and cytoplasm of cancer cell) and 11 of 86 (12.8%) showed strong staining in tumor cells and some infiltrated lymphocytes (Figure 2). Thirteen of the 15 non-cancerous tissues indicated strong expression of SATB2 and the rest two non-cancerous tissues showed moderate expression (Figure 2).

Additionally, the incidence of SATB2 protein expression in well-differentiated carcinoma was significantly higher than that in poor-differentiated tumors, and SATB2 expression was significantly related with tumor differentiation (*P* = .002) (Table 1).

**Table 1:** Correlation between the status of SATB2 and clinicopathological factors.

Variable	Cases	SATB2		*P*
		Positive	Negative	
**Age**				
<65	63	34	29	0.568
≥65	23	14	9	
**Smoking index**				
<600	40	26	14	0.110
≥600	46	22	24	
**Type**				
Supraglottic	29	79.3	72.1	0.851
Glottic	57	80.7	70.5	
**Side**				
Uniliteral	64	38	26	0.257
Biliteral	22	10	12	
**Thyroid cartilage invasion**				
No	69	41	28	0.175
Yes	17	7	10	
**T Stage**				
T1	14	11	3	0.223
T2	40	21	19	
T3	22	12	10	
T4	10	4	6	
**Lymphatic metastasis**				
No	72	43	29	0.098
Yes	14	5	9	
**Stage**				
I	13	10	3	0.209
II	37	19	18	
III	19	12	7	
IV	17	7	10	
**Histological grade**				
I	41	31	10	0.002
II	33	12	21	
III	12	5	7	
**Recurrence**				
No	59	41	15	0.000
Yes	27	7	20	

### Correlation of SATB2 Expression with Clinicopathological Features and Outcomes

The median follow-up time for overall survival was 61 months for all patients. The 2-year and 5-year overall rates for all patients were 80.2% and 71.7%, respectively. The association between SATB2 expression and the clinicopathological outcomes is shown in Table 1. SATB2 expression was significantly correlated with histological grade and tumor recurrence. Furthermore, SATB2 expression was possibly correlated with lymphatic metastasis (*P* = .098). There was no significant correlation between SATB2 expression and age, gender, smoking index, or stage (Table 1).

### Correlation of SATB2 Expression with Overall Survival

**Figure 3:**
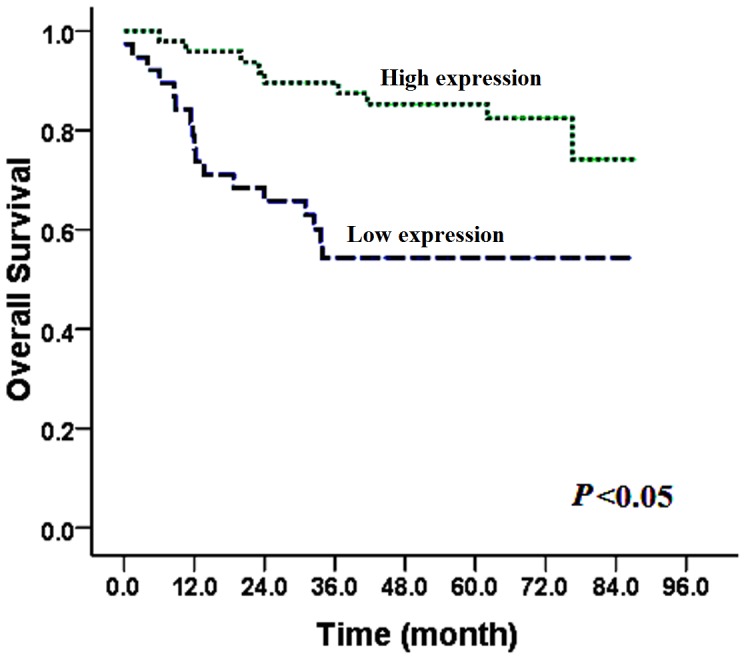
Survival curves for patients with high SATB2 expression versus low SATB2-expressing carcinoma. The 5-y overall survival rate was 85.3% in the high SATB2 protein expression group (green line) (*n*  = 48), but it was only 54.3% in the low expression group (blue line) (*n*  = 38), *P* = .002.

Among these patients, the overall survival of the patients with high SATB2 expression (5-year overall rate, 85.3%) was significantly higher than the low SATB2 expression group (5-year overall rate, 54.3%) (*P* = .002, Figure 3). Besides SATB2 expression level, age, thyroid cartilage invasion, T stage, pathological stage, histological grade, surgical margin, radiotherapy, and tumor recurrence were also significantly correlated with overall survival in univariate analysis (Table 2).

**Table 2:** Univariate survival analysis of 86 patients with laryngeal squamous cell carcinoma.

Variable	Cases	Overall survival		*P*
		2-year	5-year	
**Age (years)**				
<65	63	84.1	77.6	0.018
≥65	23	69.6	55.7	
**Smoking index**				
<600/month	40	85.0	77.3	0.652
≥600/month	46	76.1	67.1	
**Type**				
Supraglottic	29	79.3	72.1	0.851
Glottic	57	80.7	70.5	
**Side**				
Uniliteral	64	85.7	81.5	0.023
Biliteral	22	73.0	58.7	
**Thyroid cartilage invasion**				
No	69	85.5	80.9	0.000
Yes	17	58.8	35.3	
**T Stage**				
T1	14	92.9	85.1	0.006
T2	40	80.0	73.3	
T3	22	86.4	67.1	
T4	10	50.0	40.0	
**Lymphatic metastasis**				
No	72	83.3	74.7	0.174
Yes	14	64.3	56.3	
**Stage**				
I	13	92.3	92.3	0.010
II	37	81.1	78.2	
III	19	89.5	68.4	
IV	17	58.8	46.3	
**Histological grade**				
I	41	90.2	82.6	0.076
II	33	72.7	60.2	
III	12	66.7	66.7	
**Surgical margin**				
Negative	75	87.7	79.6	0.007
Positive	11	54.5	45.5	
**Radiotherapy**				
No	59	86.4	79.2	0.037
Yes	27	66.7	55.6	
**Recurrence**				
No	59	94.9	93.6	0.000
Yes	27	48.1	20.0	
**SATB2 expression**				
Negative	38	65.8	54.3	0.002
Positive	48	91.7	85.3	

The Cox proportional hazards mode was employed to evaluate the effects of the independent factors on overall survival. These factors include SATB2 expression, tumor size, lymph node metastasis, T classification, pathological stage, histological grade, surgical margin, radiotherapy and tumor recurrence. The results showed that SATB2 expression, surgical margin and tumor recurrence were recognized as independent prognostic factors of survival (Table 3). Therefore, Multivariate analysis indicated that SATB2 protein expression has a significant correlation with good prognosis of LSCC patients as an independent factor for LSCC patients.

**Table 3:** Cox regression analysis of patients with carcinoma of the larynx.

Factors	B	SE	Wald	*P*	Exp(B) with 95.0% CI
**Age**	−0.877	0.512	2.931	0.087	0.416 (0.152–1.135)
**Smoking index**	0.096	0.550	0.031	0.861	1.101 (0.375–3.233)
**Stage**	0.202	0.372	0.294	0.588	1.224 (0.590–2.538)
**Lymphatic metastasis**	1.134	0.926	1.501	0.221	3.108 (0.506–19.070)
**Histological grade**	−0.309	0.397	0.608	0.436	0.734 (0.337–1.597)
**Surgical margin**	−1.468	0.745	3.882	0.049	0.230 (0.053–0.992)
**Radiotherapy**	−0.148	0.567	0.068	0.794	0.862 (0.284–2.621)
**Recurrence**	−2.094	0.637	10.821	0.001	0.123 (0.035–0.429)
**SATB2 expression**	−1.647	0.731	5.083	0.024	0.193 (0.046–0.806)

### SATB2 Might Affect the Proliferation and Tumor Progression Ability of HEp2 Cells

**Figure 4:**
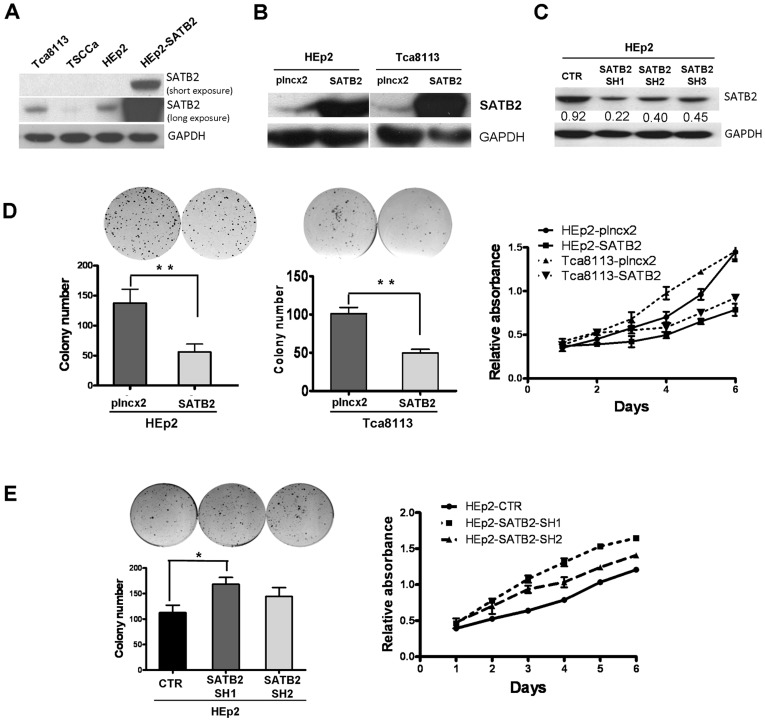
The effect of SATB2 on the proliferation and tumor progression ability of HEp2 cells. A. Western blotting analysis of SATB2 protein expression in LSCC cell line (HEp2) and HNSCC cell line (Tca 8113 and TSCCa). over-expressed SATB2 HEp2 cells (HEp2-SATB2) were used as positive control. B. Western blotting analysis of SATB2 protein expression in stably SATB2-expressed HEp2 cell line and Tca8113 cell line (HEp2-SATB2 or Tca3118-SATB2) or empty vector stable cell lines (HEp2-plncx2 or Tca3118-plncx2). C Down-regulation of SATB2 in HEp2 cells by SATB2 shRNA (HEp2-SATB2-SH1, SATB2-SH2 and SATB2-SH3). D. Colony formation assay and MTT assay of HEp2 cells and Tca8113 cells with over-expression of SATB2. (Colony formation assay: HEp2-plncx2 (vector) vs HEp2-SATB2, *P* = .0061; Tca8113-plncx2 (vector) vs Tca8113-SATB2, *P* = .0056. MTT: HEp2-plncx2 vs HEp2-SATB2, *P* = .008; Tca8113-plncx2 vs Tca8113-SATB2, *P* = .045). E. Colony formation assay and MTT assay of HEp2 cells with down-regulated SATB2. (Colony formation assay: HEp2-plncx2 vs HEp2- SATB2-SH1, *P* = .046; HEp2-plncx2 vs HEp2-SATB2-SH2, *P* = .234. MTT: HEp2-CTR (control) vs HEp2-SATB2-SH1, *P* = .001; HEp2-CTR vs HEp2- SATB2-SH2, *P* = .005).

To further investigate whether SATB2 could inhibit the proliferation and tumor progression ability of LSCC cells (HEp2) and head and neck squamous carcinoma (HNSCC) cell lines (Tca8113), we established stable HEp2 cell line and Tcs8113 cell line that expressed SATB2 (HEp2-SATB2 or Tca8113-SATB2) or empty vector (HEp2-plncx2 or Tca8113-plncx2) (Figure 4A & 4B). As shown in Figure 4A, the expression level of SATB2 was significantly increased in HEp2-SATB2 cells compared to control cells, and other cell lines (Tca8113 and TSCCa). Over-expression of SATB2 inhibited cell proliferation and malignant transforming ability of HEp2 cells and Tca8113 cells by colony formation assay and MTT assay (Figure 4D). To further investigate the effect of SATB2 in the proliferation and malignant transforming ability of LSCC cells (HEp2), we established stable HEp2 cell lines with down-regulation of SATB2 by 3 different shRNA sequences against SATB2 (HEp2-SATB2-SH1, SATB2-SH2 and SATB2-SH3). As shown in Figure 4C, the expression level of SATB2 was significantly decreased in HEp2-SATB2-SH1 and HEp2-SATB2-SH2 cells compared to control cells (HEp2-CTR). Knocking-down of SATB2 in HEp2 cells increased malignant transforming ability and cell proliferation (Figure 4E), suggesting that reduced expression of SATB2 might involve in the development of LSCC. However, we failed to examine any obvious increased number of apoptotic cells in SATB2-overexpressed cells by Annexin-V analysis (Figure S1), thus indicating that the effect of SATB2 was more possible on cell proliferation instead of apoptosis.

### Over-expression of SATB2 Inhibits the Tumor Growth in Nude Mice

**Figure 5:**
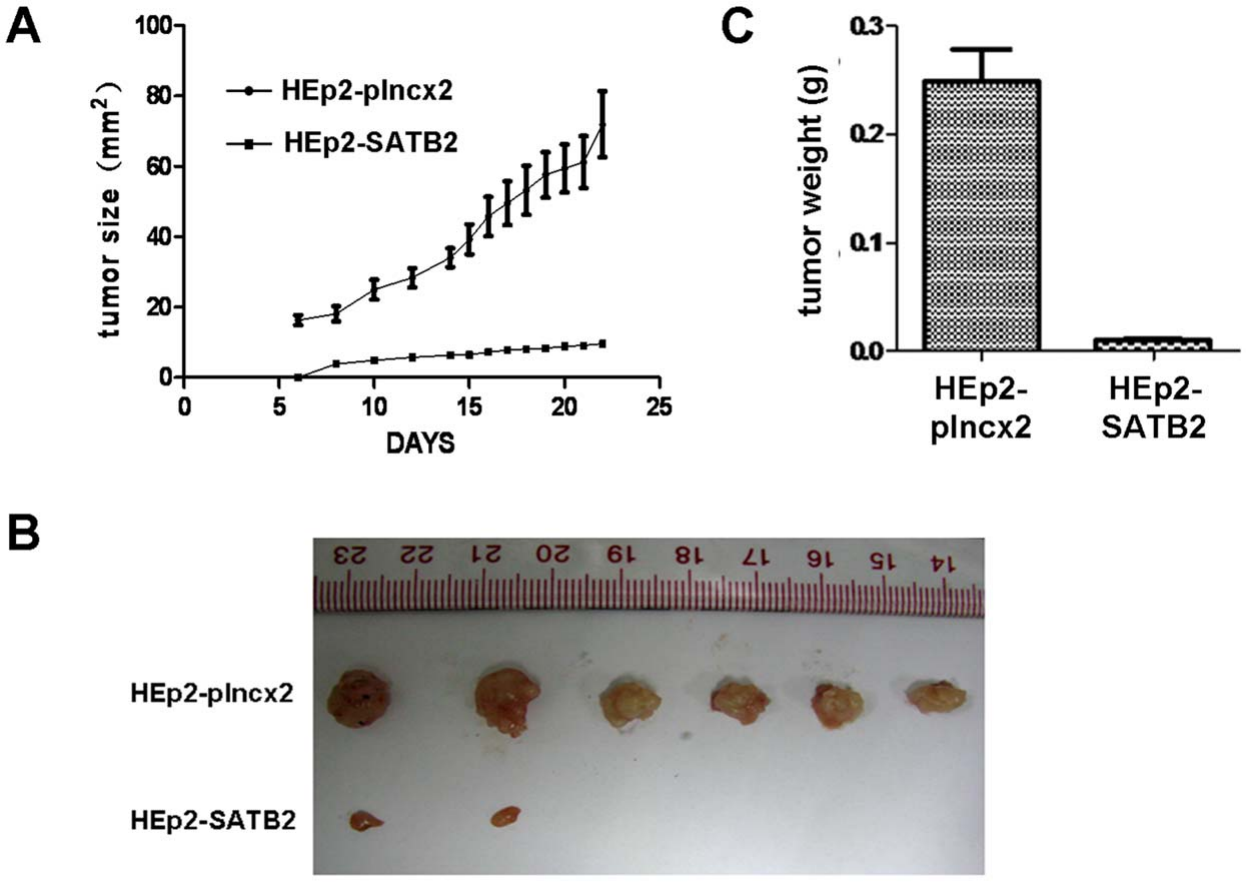
SATB2 inhibites the tumor growth in nude mice. A. Tumor growth curve of after injection of nude mice with SATB2 or vector control (plncx2) expressing HEp2 cells. (*P*<0.0001) B. The picture of tumors from nude mice with SATB2 or vector control (plncx2) expressing HEp2 cells. C. The weight of tumors from nude mice with SATB2 or vector control (plncx2) expressing HEp2 cells. (*P* = .0046).

In vivo experiment was performed to evaluate the effect of SATB2 over-expression in nude mice. As shown in Figure 5A and 5C, the growth rate and tumor weight of SATB2 tumors were found to be much lower than those with control (HEp2-plncx2). The data in Figure 5B showed that SATB2-expressing HEp2 cells formed only small tumors in 2/6 mice, whereas control cells formed tumors in 6/6 mice. The result suggested that over-expression of SATB2 inhibited tumorigenicity of HEp2 cells *in vivo*.

## Discussion

SATB2 is a novel AT-rich DNA binding protein, which regulates the gene expression through altering chromatin structure. SATB2 directly interacts with the activity of transcription factors in regulating craniofacial development, cortical neurons differentiation and osteoblast differentiation [11], [12], [13], [14], [15]. A few studies have been done in colorectal, breast and oral carcinomas to evaluate the role of SATB2 in cancer [21], [22], [23], [24], however, the results were highly controversial. Up to now, little has been known about the expression of SATB2 in LSCC tissue or cell line, although in other types of HNSCC, SATB2 seems to be an oncogene^25^. This study showed the first evidences that the status of SATB2 expression in carcinoma tissues is much lower than that in paracarcinoma tissues in LSCC, and SATB2 is an independent prognostic factor for LSCC patients.

As shown by immunohistochemical analysis, 44.2% paraffin-embedded laryngeal cancer tissues showed negative nuclear staining of SATB2, 43.0% laryngeal cancer tissues showed moderate SATB2 staining and 12.8% showed strong staining in tumor cells and some infiltrated lymphocytes, while the non-cancerous tissues presented mainly strong expression of SATB2, indicating that SATB2 might play an important role in the development and progression of LSCC. In addition, as determined by immunohistochemistry, the incidence of SATB2 protein expression in well-differentiated carcinoma was significantly higher than that in poor-differentiated tumors, suggesting that low level of SATB2 expression was related to poor tumor differentiation, and SATB2 might regulate tumor differentiation. A recent study by Wang [21] showed SATB2 protein was lower in primary colorectal cancer tissues than in their normal counterparts. Combined the results form our study and Wang’s study, SATB2 might be related to tumor differentiation. However, another study from Pantani [23] showed that the mRNA expression of SATB2 and SATB1 in human breast cancer were higher in malignant compared to normal breast tissue. Since the measurement of mRNA transcript levels may not always correlate with protein level, mRNA level might not reflect the important function of SATB2 correctly.

Additionally, we have shown that SATB2 expression was positively correlated with histological grade, and tumor recurrence, and that SATB2 expression was also a trend toward potential correlation with lymphatic metastasis (*P* = .098). There was no significant correlation between SATB2 expression and age, gender and stage. Our study suggests that lower SATB2 expression might be positively correlated with worse tumor biological features, such as rapid tumor progression and metastases, and that SATB2 plays an important role in the development and progression of LSCC. Furthermore, we have shown by multivariate analyses that patients with SATB2 protein expression in carcinoma had a better prognosis than those without SATB2 expression, and that tumor recurrence, surgical margin and the status of SATB2 protein were independent factors influencing overall survival, indicating that SATB2 is a powerful prognostic index of survival in LSCC. These findings also suggested that clinicopathological features together with detection of SATB2 in LSCC tissue could be valuable in evaluating prognosis or designing individual therapeutic policy for LSCC.

The two genes SATB1 and SATB2 are homologous genes. SATB1 may be an oncogene, which promotes the tumor growth and metastasis of breast cancer [20]. We examined the relationship between SATB2 and SATB1 in LSCC, and the results showed that the status of SATB2 protein in LSCC tissues was lower than that in paired paracarcinoma tissues, whereas the expression of SATB1 protein was much higher than that in paracarcinoma tissues, suggesting opposite effects of SATB1 and SATB2 in LSCC. However, over-expression of SATB2 in HEp2 cell line did not significantly affect the expression of SATB1 (data not shown), suggesting that SATB2 might not regulate the SATB1 expression directly. Therefore, further studies are needed to verify the interaction of SATB1 gene and SATB2 gene. In addition, our study also showed that low SATB2 expression is a prognostic factor indicating poor survival, suggesting that the reduced SATB2 level might be a risk factor for LSCC. A recent study showed that low expression of SATB2 was correlated with poor prognosis, tumor invasion, metastasis and Dukes' classification in colorectal cancer [21], further confirming the decreased level of SATB2 is a powerful prognostic factor in cancer.

Additionally, we investigated whether SATB2 could affect the proliferation rate and tumorigenicity of LSCC cells (HEp2) and additional head and neck squamous carcinoma (HNSCC) cell line Tca8113 and found that over-expression of SATB2 inhibited cell proliferation and colony formation ability of HEp2 cells and Tca8113 cells. Over-expression of SATB2 inhibited tumorigenicity of HEp2 cells in nude mice. However, over-expression of SATB2 could not induce apoptosis in HEp2 cells. These results suggested SATB2 might be involved in the development of LSCC as a tumor suppressor by inhibiting cell proliferation and tumorigenicity.

SATB2 expression status, combined with clinicopathological features and other biomarkers of LSCC, may be useful to stratify patients for individual treatment, such as those of adjuvant chemotherapy, or radiotherapy. Further investigation in other patient population or group is required to verify these hypotheses. Since the number of the cases in this study was not too big, the relationship between SATB2 expression and metastases still requires to be evaluated. A recent study showed that SATB2 might promote chemoresistance by augmenting DNp63a engagement to p53-family responsive elements in oral tissues including the tongue, mouth floor, buccal mucosa and gingival [24], however the study was about oral cancer and the case scale was small. Therefore, further studies are needed to clarify the mechanisms by which SATB2 is involved in the development and progression of LSCC as a tumor suppressor and its exact role in the regulation of chromosome instability in LSCC.

In conclusion, this study showed the first evidences of the expression and clinical significance of SATB2 in LSCC, suggesting that SATB2 might involve in the development and progression of LSCC as a tumor suppressor, and thereby may serve as a valuable prognostic marker for LSCC patients.

## Supporting Information

Figure S1 **Annexin-V flow cytometry analysis of HEp2 cells with over-expression of SATB2 gene.** Annexin-V flow cytometry analysis of stably SATB2-expressed HEp2 cell line (HEp2-SATB2) or empty vector stable cell lines (HEp2-plncx2). HEp2 cells were grown in suspension culture at poly-HEMA coated dishes (HEp2-suspension).
(TIF)
