# Supplementary figures and images for: Decreased Expression of SATB2: A Novel Independent Prognostic Marker of Worse Outcome in Laryngeal Carcinoma Patients
Source: PLoS One. 2012 Jul 16;7(7):e40704. doi: 10.1371/journal.pone.0040704 (PMC3398043; doi:10.1371/journal.pone.0040704)

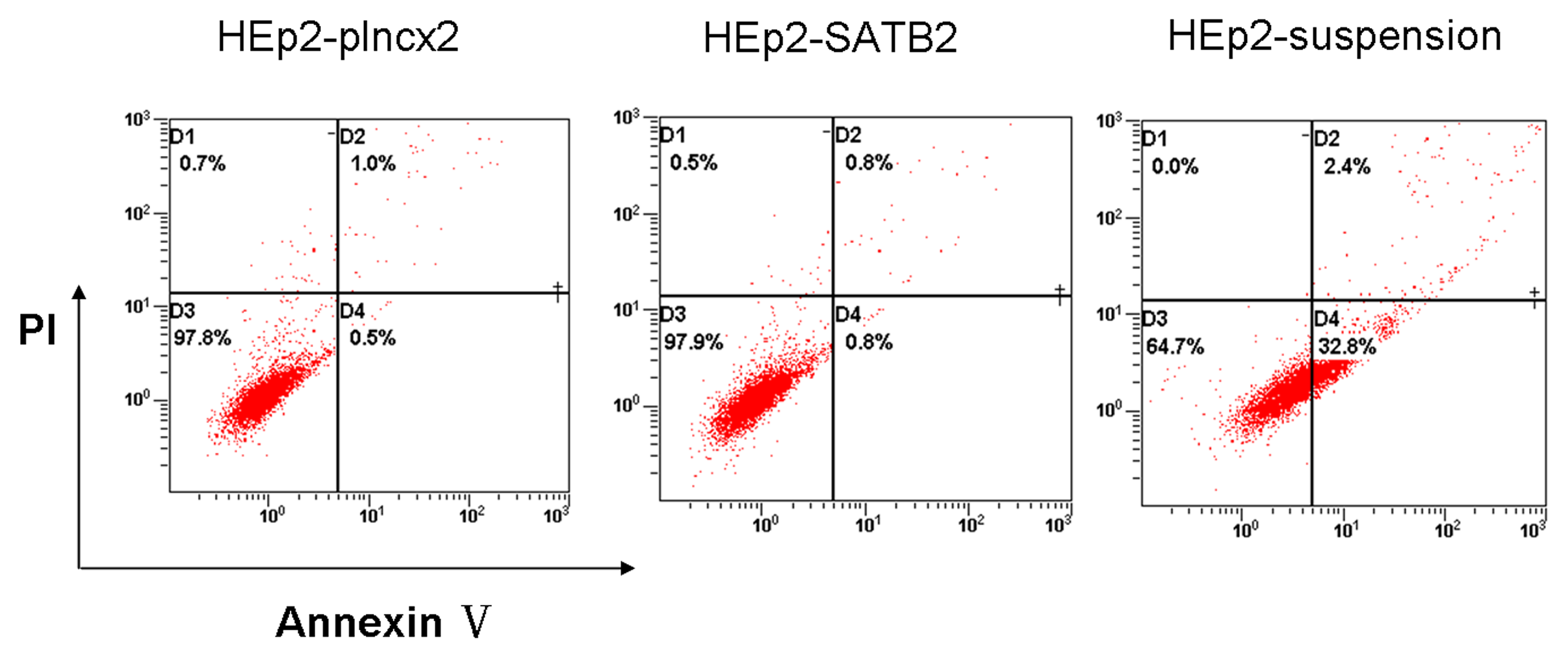

Supplement: Figure S1 — Annexin-V flow cytometry analysis of HEp2 cells with over-expression of SATB2 gene. Annexin-V flow cytometry analysis of stably SATB2-expressed HEp2 cell line (HEp2-SATB2) or empty vector stable cell lines (HEp2-plncx2). HEp2 cells were grown in suspension culture at poly-HEMA coated dishes (HEp2-suspension). (TIF) [file pone.0040704.s001.tif]
